# Effects of plantar fascia on first metatarsophalangeal joint stress in different foot types

**DOI:** 10.1186/1757-1146-7-S1-A58

**Published:** 2014-04-08

**Authors:** Rajshree Mootanah, Khadija Saoudi, Joel Mazella, Antoine Truchetet, Jonathan Deland, Scott Ellis, Josh Baxter, Howard J Hillstrom

**Affiliations:** 1 Anglia Ruskin University, Chelmsford, Essex, UK; 2 Universite de Lorraine, Nancy, France; 3 Ecole des mines d'Albi-Carmaux, Albi, France; 4 Hospital for Special Surgery, NY, USA

## 

Osteoarthritis (OA) is the leading cause of disability in older adults [1] and 1^st^ metatarsophalangeal joint (MTPJ) OA, is the most common form of OA in the foot [2]. Many foot pathologies are of a biomechanical nature and often associated with one foot type over another [3,4]. OA is postulated to result from elevated joint stress. However, the link between stress distribution in the 1^st^ MTPJ and different foot types is not well understood. Furthermore the tension band effect of the plantar fascia upon 1^st^ MTP joint function is also not well understood.

A high resolution 7 Tesla MRI was used to create a geometrically accurate 3D model of the 1^st^ MTPJ using Mimics v14 imaging software. To simulate rectus, planus and cavus feet, 1^st^ metatarsal declination angles of 20.2°, 10.1° and 30.7° were constructed. Plantar fascia material properties were altered. Physiological material properties and boundary conditions were applied to solve for stress, using ABAQUS. The ligaments were simulated by linear spring elements. The base of the first metatarsal bone was mechanically grounded in this model. Plantar loading conditions were applied, based on plantar pressure data collected from different foot types.

**Figure 1:**
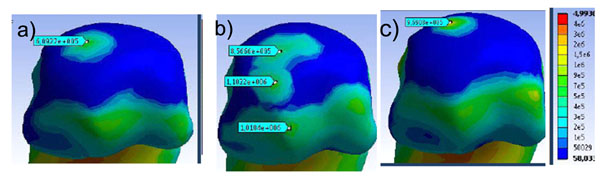
1^st^ MTPJ stress a) rectus, b) planus, and c) cavus foot types

**Figure 2:**
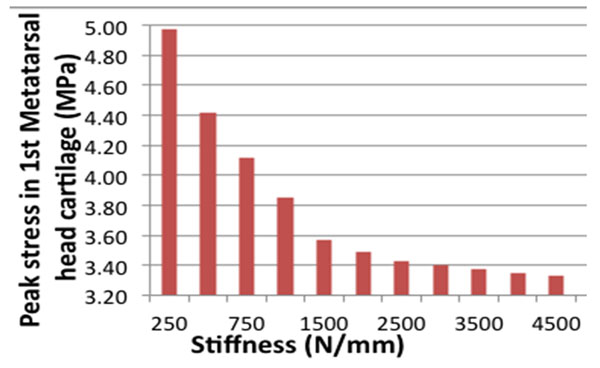
Fascia stiffness effects

Results of our static 3D FE model (Figure 1) during mid-stance of gait showed peak stresses in the distal 1st MTPJ cartilage of 0.61 MPa, 0.97 MPa and 1.10 MPa for the rectus, cavus and planus foot type, respectively (Figure 1). First MTP joint stress is largest for planus and cavus foot types and least for well-aligned rectus feet. Global foot alignment affected the magnitude and location of peak stress within the joint. Peak stress decreases as the k increases suggesting that the plantar fascia plays an important ‘tension band’ effect (Figure 2). Future research should develop methods of tuning plantar fascia material properties to represent a specific patient.
